# Parsimonious genotype by environment interaction covariance models for cassava (*Manihot esculenta*)

**DOI:** 10.3389/fpls.2022.978248

**Published:** 2022-09-21

**Authors:** Moshood A. Bakare, Siraj Ismail Kayondo, Cynthia I. Aghogho, Marnin D. Wolfe, Elizabeth Y. Parkes, Peter Kulakow, Chiedozie Egesi, Jean-Luc Jannink, Ismail Yusuf Rabbi

**Affiliations:** ^1^Plant Breeding and Genetics Section, School of Integrative Plant Science, College of Agriculture and Life Sciences, Cornell University, Ithaca, NY, United States; ^2^International Institute of Tropical Agriculture, Ibadan, Nigeria; ^3^West Africa Centre for Crop Improvement, University of Ghana, Legon, Ghana; ^4^Department of Crop, Soil and Environmental Sciences, College of Agriculture, Auburn University, Auburn, AL, United States; ^5^National Root Crops Research Institute (NRCRI), Umudike, Umuahia, Nigeria; ^6^USDA-ARS, Robert W. Holley Center for Agriculture and Health, Ithaca, NY, United States

**Keywords:** factor analytic model, genotype-by-environment interaction, partial least squares regression, variance structure, factor loadings, genotypic scores, hybrid relationship matrix, environmental covariables

## Abstract

The assessment of cassava clones across multiple environments is often carried out at the uniform yield trial, a late evaluation stage, before variety release. This is to assess the differential response of the varieties across the testing environments, a phenomenon referred to as genotype-by-environment interaction (GEI). This phenomenon is considered a critical challenge confronted by plant breeders in developing crop varieties. This study used the data from variety trials established as randomized complete block design (RCBD) in three replicates across 11 locations in different agro-ecological zones in Nigeria over four cropping seasons (2016–2017, 2017–2018, 2018–2019, and 2019–2020). We evaluated a total of 96 varieties, including five checks, across 48 trials. We exploited the intricate pattern of GEI by fitting variance–covariance structure models on fresh root yield. The goodness-of-fit statistics revealed that the factor analytic model of order 3 (FA3) is the most parsimonious model based on Akaike Information Criterion (AIC). The three-factor loadings from the FA3 model explained, on average across the 27 environments, 53.5% [FA (1)], 14.0% [FA (2)], and 11.5% [FA (3)] of the genetic effect, and altogether accounted for 79.0% of total genetic variability. The association of factor loadings with weather covariates using partial least squares regression (PLSR) revealed that minimum temperature, precipitation and relative humidity are weather conditions influencing the genotypic response across the testing environments in the southern region and maximum temperature, wind speed, and temperature range for those in the northern region of Nigeria. We conclude that the FA3 model identified the common latent factors to dissect and account for complex interaction in multi-environment field trials, and the PLSR is an effective approach for describing GEI variability in the context of multi-environment trials where external environmental covariables are included in modeling.

## Introduction

Cassava (*Manihot esculenta* Crantz) is one of the most essential food-security crops in developing countries, particularly in tropical and subtropical regions (Tumuhimbise et al., 2014; Nduwumuremyi et al., 2017). It is a crop grown predominantly by smallholders for subsistence due to its adaptability to survive in drought-prone areas under marginal conditions where other crops may not thrive (Egesi et al., 2007; Sayre et al., 2011). Though cassava grows well in diverse environments, its yield production differs among the genotypes and environments. This difference is due to inbuilt genetic properties, environmental conditions, and genotype-by-environment interaction (Falconer, 1996).

It has long been recognized that phenotypic expression of genotypes is much influenced by environmental conditions (Meyer, 2009). This can result in heterogeneity of variability and different ranking of genotypes performance in different environments, a phenomenon described as genotype-by-environment interaction (GEI). The phenotypic panel for evaluating GEI is often called a multi-environment trial (MET). Traditionally, the resulting empirical data from METs are often analyzed using classical statistical methods (Bakare et al., 2022). These methods include ANOVA, fixed linear bilinear model such as additive main effect and multiplicative interaction (AMMI) model (Gauch and Zobel, 1997; Gauch, 2016) and site regression (SREG) or genotype main effect and genotype-by-environment (GGE) model (Yan et al., 2000), and linear regression type model like Finlay and Wilkinson (1963). These classical analyses are inefficient in handling unbalanced datasets that often arise in METs (Bakare et al., 2022), resulting in unreliable estimates of genetic effects.

Linear mixed models that include fixed and random effects are increasingly used to analyze MET in a plant breeding program (Piepho, 1998b; Smith et al., 2005; Burgueño et al., 2008). These models are centered around a factor analytic (FA; Piepho, 1997, 1998a) form of genetic variance–covariance structure. Factor analytic structures have been reported to be more parsimonious and flexible than other variance–covariance structures (Crossa, 2012), allowing the estimation of a fewer number of parameters in comparison to unstructured (US) variance–covariance model (Smith et al., 2001a,b; Kelly et al., 2007). Graphical tool like heatmaps of estimated genetic correlation across the testing environments (Cullis et al., 2014; Smith et al., 2015) resulting from factor analytic model can be used to make inferences about GEI, adaptability and stability of genotypes (Oliveira et al., 2020). Also, the factor loadings which are environmental effects in the latent factors can be correlated with external environmental covariables such as solar radiation, temperature, precipitation, relative humidity, wind speed and others, to examine the pattern of genotypic response across environments. The measure of these external environmental covariables in different developmental phases of year-long growth period crops such as cassava will result in many predictor variables that are highly correlated. The use of ordinary least squares regression model to quantify the relationship between dependent variable(s) and predictor variables is not adequate due to multicollinearity problem. In this scenario, partial least squares regression (Aastveit and Martens, 1986; Talbot and Wheelwright, 1989; Vargas et al., 1998) can be used to determine which among these environmental covariables influence GEI of fresh root yield.

To date, no implementation of the FA model in the genetic assessment of cassava clones has been reported in Africa nor environmental covariables driving GEI have been explored. However, few studies have been reported to explore GEI in cassava and these studies were conducted in few environments using ANOVA, AMMI (Dixon and Ssemakula, 2007; Jiwuba et al., 2020) and GGE (Akinwale et al., 2011) for analyses. This study examines the utility of variance–covariance structure models and partial least squares regression to: (i) identify optimal variance–covariance structure model that captured GEI and stable genotypes; (ii) identify mega environments, and (iii) identify key environmental covariables that explained GEI for fresh root yield.

## Materials and methods

### Clonal material and field experimental design

**Figure 1:**
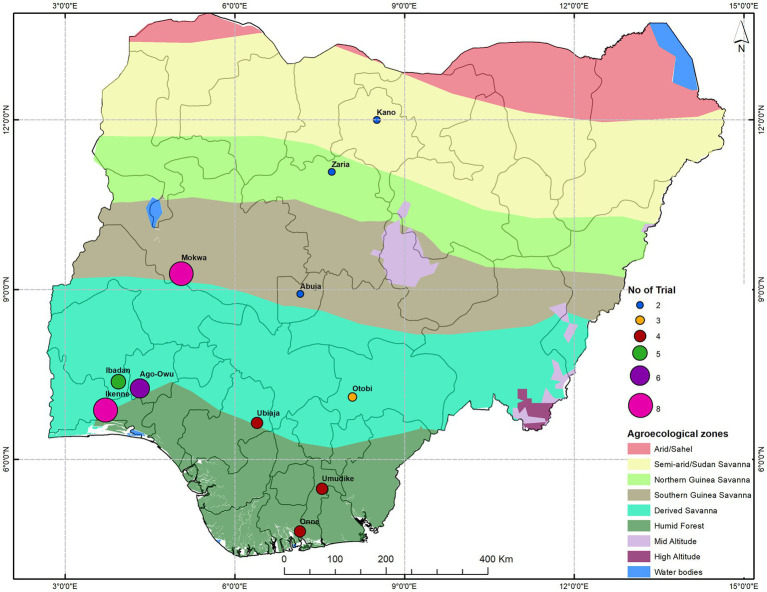
A map of Nigeria showing the trial geographical locations across agro-ecological zones.

This study used 48 uniform yield trials in three sets named setA, setB, and setC with, respectively, 36, 36, and 34 clones each. A total of 96 clones were evaluated, corresponding to 91 breeding lines and five checks common across sets. These clones were derived from elite X elite crosses as part of a genomic recurrent breeding program. Prior to this field evaluation, they were assessed for susceptibility to cassava mosaic disease (CMD), cassava bacteria blight (CBB), early vigor, and other agronomic traits of interest in earlier evaluation stages. The clones in the UYT were high yielding materials that have passed several stages of field evaluation and selection to eliminate disease susceptible clones. The clones were evaluated in UYT trials in 11 locations across different agro-ecological zones in Nigeria (Figure 1) over four growing seasons (2016–2017, 2017–2018, 2018–2019, and 2019–2020).

Each trial was established as a Randomized Complete Block Design (RCBD) with two or three replicates. The experimental plot consisted of six rows of length 5.6 m with an inter-row spacing of 1 m and intra-row spacing of 0.8 m and only the interior 20 plants (4 m × 4 m) were harvested. Across the full dataset, there were 28 environments (location by year combinations) and a total of 4,575 plots, varying in number across the testing environments from 72 (Onne20) to 318 (Ikenne18 and Mokwa18; Table 1). The trait of interest in this study was fresh root yield (t/ha).

**Table 1:** Summary of number of trials, cassava clones, plots, blocks and mean fresh root yield (FYLD) per environment.

Environment	Trial	Clones	Plots	Blocks	FYLD (*t*/ha)
Abuja20	2	67	144	4	26.0
Ago-Owu18	2	67	216	6	34.0
Ago-Owu19	2	67	216	6	28.7
Ago-Owu20	2	67	144	4	41.0
Ibadan18	1	33	99	3	36.8
Ibadan19	2	67	216	6	39.9
Ibadan20	2	67	144	4	26.5
Ikenne17	1	34	102	3	37.0
Ikenne18	3	96	318	9	34.1
Ikenne19	2	67	216	6	17.4
Ikenne20	2	67	144	4	41.9
Kano19	2	67	216	6	15.2
Mokwa17	1	34	102	3	22.4
Mokwa18	3	96	318	9	31.7
Mokwa19	2	67	216	6	20.9
Mokwa20	2	67	144	4	18.6
Onne18	1	34	102	3	28.9
Onne19	2	67	216	6	16.9
Onne20	1	36	72	2	13.0
Otobi18	1	34	102	3	25.9
Otobi19	2	67	216	6	41.6
Ubiaja17	1	34	102	3	33.2
Ubiaja18	1	34	102	3	27.6
Ubiaja20	2	67	144	4	15.7
Umudike17	1	34	102	3	24.2
Umudike18	1	34	102	3	21.3
Umudike19	2	67	216	6	31.9
Zaria20	2	67	144	4	13.7

### Genotype and pedigree relationship matrices

Following a modified cetyltrimethyl ammonium bromide (CTAB) method, we extracted high-quality genomic DNA from freeze-dried cassava leaf samples (Dellaporta et al., 1983). The Nanodrop spectrophotometer operating at an absorbance of 260 nm qualified and quantified the extracted DNA before genotyping. The genotyping-by-sequencing (GBS) approach generated a dense genome-wide single nucleotide polymorphism (SNP) dataset as described by Elshire et al. (2011). The ApeKI enzyme reduced genome complexity through restriction digestion, preparing genomic fragments for GBS (Hamblin and Rabbi, 2014). Sequence alignment of the resultant sequence tags was done using the cassava Version 6 genome as a reference (Prochnik et al., 2012). Alignment was followed by the SNP calling step using TASSEL GBS pipeline V4 (Glaubitz et al., 2014). All SNP calls below five reads were masked before imputation using Beagle V4.1 (Browning and Browning, 2016). After imputation, 73,599 biallelic SNP markers with an estimated allelic r-squared value (AR^2^) of more than 0.3 were retained for subsequent analyses. Data quality control was carried out on the SNP dataset using the *qc.filtering()* function in the *ASRgenomics* library (Gezan et al., 2021) prior to downstream analyses. The filtering criteria included: (i) removal of SNPs with minor allele frequency (MAF) below 0.05, (ii) removal of individuals whose proportion of missing values was equal or above 20% (call rate 0.2), and (iii) removal of SNPs whose proportion of missing values equal or larger than 20%, retaining 68,279 SNPs in total. However, the available SNP marker data was only available for 81 clones. Thus, we also used pedigree data on 123 individuals out of which 27 individuals were dropped to have a pedigree-based relationship matrix of dimension 96 × 96 for the phenotyped cassava clones. The SNP marker set was used in the derivation of a genomic relationship matrix (GRM) and combined with the pedigree relationship matrix to produce a hybrid relationship matrix (H).

The pedigree-based additive numerator relationship matrix (A-matrix) was constructed following the recursive method presented in Mrode (2014) and was estimated using the *Amatrix()* function of the *AGHmatrix* library (Amadeu et al., 2016). The marker-based relationship matrix (*G*) and its inverse (*G^−1^*) were estimated from SNP marker data using the *G.matrix()* and *G.inverse()* functions of the *ASRgenomics* library (Gezan et al., 2021), respectively.

The H-matrix relates all individuals through the A-matrix but integrates the additional information provided by the G-matrix. The main notion is to replace entries of the A-matrix by the corresponding entries of G-matrix and then adjust the remaining relationships accordingly. Martini et al. (2018) defined matrix H as


(1) $$H=A+\left[\begin{array}{cc} A_{12}A_{22}^{-1}\left(G-A_{22}\right)A_{22}^{-1}A_{21} & A_{12}A_{22}^{-1}\left(G-A_{22}\right)\\ \left(G-A_{22}\right)A_{22}^{-1}A_{21} & \left(G-A_{22}\right) \end{array}\right]$$

where individuals are partitioned into those without (group 1) versus with (group 2) marker data. Therefore, A_11_ contains cells of the A-matrix with relationships within the first group, A_12_ and A_21_ contain cells of the A-matrix with relationships between the individuals of the two groups, and A_22_ contains cell of the A-matrix with relationships within the second group. In this definition of the H-matrix, the inner group pedigree relationship of second group was replaced by the G-matrix indicating that H_22_ = G. The term $A_{12}A_{22}^{-1}\left(G-A_{22}\right)$ adapts the relationships within the first group and the relationships between the two groups in accordance to the changed relationships within second group to generate a positive semi-definite and valid covariance structure (Martini et al., 2018).

Since many analyses use the inverse of H that allows for simpler computations, Eq. (1) is often written in terms of its inverse (Misztal et al., 2010; Martini et al., 2018) as


(2) $$H^{-1}=A^{-1}+\left[\begin{array}{cc} 0 & 0\\ 0 & \left(G^{-1}-A_{22}^{-1}\right) \end{array}\right]$$

where G^−1^ is the inverse of genomic relationship matrix and $A_{22}^{-1}$ is the inverse of the pedigree-based relationship matrix for genotyped individuals. An approach to combine the A-matrix and G-matrix optimally is implemented by specifying a parameter λ as described by Martini et al. (2018). We used a λ value of 0.9, where λ scales the difference between genomic and pedigree-based information (Misztal et al., 2010), leading to express Eq. (2) as


(3) $$H^{-1}=A^{-1}+\left[\begin{array}{cc} 0 & 0\\ 0 & \lambda \left(G^{-1}-A_{22}^{-1}\right) \end{array}\right]$$

The G matrix was derived following (VanRaden, 2008):


(4) $$G=\frac{\left(M-P\right){\left(M-P\right)}^{`}}{2\sum_{j=1}^{m}p_{j}\left(1-p_{j}\right)}$$

where *M* is an allele-sharing matrix with *m* columns (*m* = total number of markers) and *n* rows (*n* = total number of genotyped individuals), and *P* is a matrix containing, in each column, the frequency of second allele (*p*_j_) expressed as 2*p*_j_. M_ij_ was 0 if the genotype of individual *i* for SNP *j* was homozygous *aa*, 1 if heterozygous *Aa*, or 2 if the genotype was homozygous *AA*. We note that because all columns of matrix M from which G is constructed are centered, G should not be invertible (contrary to its use in Equation 3). In practice, a number of options are available for matrices that are close to being positive definite (Tier et al., 2015) and we did not encounter difficulty in using the H-matrix described here.

### Environmental covariables

Weather data was exploited to identify the potential environmental covariates that influence differential response of the clonal lines across the testing environments. According to each trial’s location and growth dates, weather data were collected from the database of the National Aeronautics and Space Administration Prediction of Worldwide Energy Resource (NASA POWER) project.^1^ The data included: minimum temperature (°C), maximum temperature (°C), temperature range (°C), precipitation (mm), relative humidity (%), wind speed (m/s), solar radiation (W/m^2^), surface soil wetness (%), root zone soil wetness (%), and profile soil moisture (%) for the whole crop growth cycle, i.e., from planting to harvesting of each field trial.

## Statistical models

### Single trial analysis and data quality control

Before formal analysis, the observed agronomic traits’ empirical distribution was visualized across the trials using boxplots and the *ggplot2* package (Wickham, 2016) in R (R Core Team, 2018). The statistical analysis of individual trials was carried out in a linear mixed model framework and the variance components were estimated by restricted maximum likelihood. The univariate linear mixed model fitted was:


(5) $$y=\mu +X_{1}r+p\beta +Z_{1}g+\epsilon$$

where *y* is the (*n* × 1) vector of observed phenotypic values, in which *n* is the number of observations in the trial; μ is the intercept (overall mean); *r* is the (*r* × 1) vector of fixed effect of replicates with its associated incidence matrix *X_1_* of dimension *n* × *r*; *p* denotes the proportion of plant stands harvested as a covariate (e.g., if 28 stands were planted, but only 21 harvested, *p* = 0.75); β is a regression coefficient relating *p* and *y*; *g* is the (*g* × 1) vector of random effect of genotype with its associated design matrix Z_1_ of dimension *n* × *g*, and ϵ is a residual term which is assumed to follow a Gaussian distribution, $\epsilon \sim N\left(0,\;I_{n}\sigma_{\epsilon }^{2}\right)$.

The quality of each trial was assessed by calculating the coefficient of variation (CV), broad-sense heritability (H^2^) on an entry-mean basis, and experimental accuracy (Ac) proposed by Mrode (2014) using the following equations: $CV\left(\%\right)=\left({\hat{\sigma }}_{e}/\bar{y}\right)\times 100$, $H^{2}={\hat{\sigma }}_{g}^{2}/\left({\hat{\sigma }}_{g}^{2}+{\hat{\sigma }}_{e}^{2}/r\right)$, and $Ac=\sqrt{\left(1-PEV/{\hat{\sigma }}_{g}^{2.}\right)}$where ${\hat{\sigma }}_{e}$ is the estimated residual standard deviation, $\bar{y}$ is the estimate of the overall mean for an agronomic trait; ${\hat{\sigma }}_{g}^{2}$ is the estimated genetic variance, ${\hat{\sigma }}_{e}^{2}$ is the estimated error variance, *r* is the number of replicates, and PEV is the average of prediction error variance. A trial was removed from a combined analysis based on any of these conditions: The thresholds of CV above 40.5%, H^2^ below 0.14 or Ac below 0.40.

### Variance–covariance structure models

Before fitting the models, we examined the degree of clone connectivity between pairs of environments (Supplementary Figure 1). This was to have a prior knowledge of the amount of information for estimating a genetic covariance between pairs of environments. Seven variance–covariance structure models were fitted to describe and explore the pattern of GEI. The analysis was carried out using the software ASREML-R version 4.0 (Butler et al., 2017) within the R statistical environment (R Core Team, 2018). This package fits linear mixed models allowing heterogeneity of genetic and error variances across environments where the variance component is estimated using the average information algorithm (Gilmour et al., 1995).

The variance structure models were fit to the data in one-stage analyses using the following linear mixed model:


(6) y=μ+e+set(e)+pβ+r(sete)+g+ϵ

where *y,* μ, *p* and β were as defined in the previous equation, *e* is the (*s* × 1) vector of fixed effect of the environment where *s* is the number of environments; *set(e)* is the fixed effect of the trial set nested within the environment; *r(set e)* is the fixed replicate effect nested with set and environment; *g* is random effect of genotype nested within environments: g = $\left[g_{1}^{T},g_{2}^{T},\ldots ,g_{s}^{T}\right],$ where $g_{j}^{T}$ is the vector of genotypic effects in environment *j* with its associated hybrid relationship matrix (*H*); $g\sim N\left(0,\Sigma \right)$ (see below for the specification of Σ); and ϵ is a residual term that is heterogeneous across the testing environments.

We partitioned the total genetic effects (g) into additive (a) and non-additive (i) components (Oakey et al., 2007) which are assumed to be independent such that a ~ N $\left(0,\sigma_{a}^{2}H\right)$, i ~ N $\left(0,\sigma_{i}^{2}I\right)$ and *I* is the identity matrix. The non-additive component captures other effects such as dominance, epistasis, and residual additive effects which are not captured by *H*-matrix. We used an identity matrix to capture that residual after fitting the non-additive effect. This necessitated the scaling of the hybrid matrix associated with additive genetic effect by multiplying main additive genetic and interaction variance matrices by the average of diagonal element of H-matrix which was estimated to be approximately 0.97, closely corresponding to the diagonal element of an identity matrix.

### Diagonal variance structure model

We fitted a diagonal variance (DIAG) model as a baseline. This variance–covariance model postulates independence of genetic effects among environments. Being an environment or trial-specific model, if a trial is found to have no genetic variance (variance estimated to be zero), such trial will be excluded from the analysis. The estimates from this model are often used as a starting values when fitting a more complex model like the factor analytic (FA) model. The covariance structure is of the form (assuming four environments):


(7) $$\sum =\left[\begin{array}{cccc} \sigma_{1}^{2} & 0 & 0 & 0\\ 0 & \sigma_{2}^{2} & 0 & 0\\ 0 & 0 & \sigma_{3}^{2} & 0\\ 0 & 0 & 0 & \sigma_{4}^{2} \end{array}\right]⊗H$$

where the main diagonal elements are the unique genetic variances within environments. For example, $\sigma_{1}^{2}$ is the genetic variance within an environment 1; and *H* is the hybrid relationship matrix combining pedigree and genomic relationship matrices to account for the relatedness among the cassava clones and same for other models described below.

### Compound symmetry model

The compound symmetry (CS) is the most restrictive variance–covariance model. It postulates homogeneity across environments of genetic variance $\left(\sigma_{g}^{2}+\sigma_{ge}^{2}\right)$ and uniform covariance between any pair of environments $\left(\sigma_{g}^{2}\right).$ Note that this variance–covariance model is equivalent to estimating a fixed genotype-by-environment-interaction variance. Its covariance structure is of the form


(8) $$\sum =\left[\begin{array}{cccc} \;\sigma_{g}^{2}+\sigma_{ge}^{2} & \sigma_{g}^{2} & \sigma_{g}^{2} & \sigma_{g}^{2}\\ \sigma_{g}^{2} & \sigma_{g}^{2}+\sigma_{ge}^{2} & \sigma_{g}^{2} & \sigma_{g}^{2}\\ \sigma_{g}^{2} & \sigma_{g}^{2} & \sigma_{g}^{2}+\sigma_{ge}^{2} & \sigma_{g}^{2}\\ \sigma_{g}^{2} & \sigma_{g}^{2} & \sigma_{g}^{2} & \sigma_{g}^{2}+\sigma_{ge}^{2} \end{array}\right]⊗H$$

### Compound symmetry heterogeneous model

The compound symmetry heterogeneous (CSH) is an extension of the CS model which postulates a uniform correlation between any pair of environments but heterogeneity across environments of genetic variance and covariance. Its covariance structure is of the form


(9) $$\sum =\left[\begin{array}{cccc} \;\sigma_{1}^{2} & \rho \sigma_{1}\sigma_{2} & \rho \sigma_{1}\sigma_{3} & \rho \sigma_{1}\sigma_{4}\\ \rho \sigma_{1}\sigma_{2} & \sigma_{2}^{2} & \rho \sigma_{2}\sigma_{3} & \rho \sigma_{2}\sigma_{4}\\ \rho \sigma_{1}\sigma_{3} & \rho \sigma_{2}\sigma_{3} & \sigma_{3}^{2} & \rho \sigma_{3}\sigma_{4}\\ \rho \sigma_{1}\sigma_{4} & \rho \sigma_{2}\sigma_{4} & \rho \sigma_{3}\sigma_{4} & \sigma_{4}^{2} \end{array}\right]⊗H$$

where the main diagonal elements were as in Eq. (7), and off-diagonal elements are unique genetic covariances between pairs of environments. For example, $\rho \sigma_{1}\sigma_{2}$ is the genetic covariance between environment 1 and 2 in which ρ is the uniform genetic correlation between pairs of environments, and $\sigma_{1}$ and $\sigma_{2}$ are genetic standard deviations of environment 1 and 2, respectively.

### Unstructured model

The unstructured (US) model is the least restrictive variance–covariance model, and describes the covariance based on the assumption of heterogeneity of variance within environments and unique covariance between any two environments. As the number of environments (denoted by *s*) increases, it requires a high number of parameters (p=s(s+1)/2) resulting in increased computational demand and instability. Therefore, it is rarely used in modeling GEI in the analysis of MET data with a large number of environments. We give this model here for completeness though we were not able to fit it to our data. Its covariance structure is of the form


(10) $$\sum =\left[\begin{array}{cccc} \sigma_{1}^{2} & \sigma_{12} & \sigma_{13} & \sigma_{14}\\ \sigma_{21} & \sigma_{2}^{2} & \sigma_{23} & \sigma_{24}\\ \sigma_{31} & \sigma_{32} & \sigma_{3}^{2} & \sigma_{34}\\ \sigma_{41} & \sigma_{42} & \sigma_{43} & \sigma_{4}^{2} \end{array}\right]⊗H$$

where $\sigma_{ij}=\sigma_{ji}$ (the matrix was symmetric), the main diagonal elements were as in Eq. (7), and off-diagonal elements represent unique covariances between pairs of environments.

### Factor analytic model

The factor analytic (FA) model is the random effect analogue of AMMI model (Smith and Cullis, 2018) for describing the structure of GEI. It identifies latent (unobserved) common factors that explain GEI while allowing each environment to have a specific variance for effects not explained by the common factors. The FA model provides a parsimonious approximation to the unstructured variance–covariance model (Kelly et al., 2007) but it requires fewer parameters. The model expresses $g_{ij},$ the random effect of *i*th genotype in the *j*th environment as:


(11) $$g_{ij}=\overset{t}{\underset{k=1}{\sum }}\lambda_{jk}f_{ik}+\delta_{ij}$$

where $\lambda_{jk}$ is the loading for latent factor *k* in the *j*th environment (environmental potentiality); $f_{ik}$ is the score or sensitivity of the *i*th genotype (genotypic sensitivity) for latent factor *k* related to the *j*th environment in $\lambda_{jk}$; and $\delta_{ij}$ is the residual term representing lack of fit to the model. Thus, the FA model expresses the random effect of *i*th genotype in the *j*th environment as a linear function of latent factors $\lambda_{jk}$ with random sensitivity $f_{ik}$ for *k* = 1, 2, …, *t* plus an error term $\delta_{ij}$.

The specification of FA model in a covariance form is


(12) $$G=\left(\Lambda \Lambda^{T}+\psi \right)⊗H=FA\left(k\right)+H$$

where


$$\begin{array}{c} \Lambda \Lambda^{T}+\psi =\left[\begin{array}{cccc} \lambda_{11} & \lambda_{12} & \cdots & \lambda_{1k}\\ \lambda_{21} & \lambda_{22} & \cdots & \lambda_{2k}\\ \vdots & \vdots & \ddots & \vdots \\ \lambda_{s1} & \lambda_{s2} & \cdots & \lambda_{sk} \end{array}\right]\left[\begin{array}{cccc} \lambda_{11} & \lambda_{21} & \cdots & \lambda_{s1}\\ \lambda_{12} & \lambda_{22} & \cdots & \lambda_{s2}\\ \vdots & \vdots & \ddots & \vdots \\ \lambda_{1k} & \lambda_{2k} & \cdots & \lambda_{sk} \end{array}\right]\\ +\left[\begin{array}{cccc} \Psi_{1} & 0 & \cdots & 0\\ 0 & \Psi_{2} & \cdots & 0\\ \vdots & \vdots & \ddots & \vdots \\ 0 & 0 & \cdots & \Psi_{s} \end{array}\right] \end{array}$$

where Λ is a *s* × t matrix of loadings, with the k^th^ column containing the environment loadings for the k^th^ latent factor (*k* = 1, 2, …, *t*), and Ψ is an *s* × *s* diagonal matrix with a specific variance for each environment. As above, *s* is the number of environments.

The FA model can be also taken to be a linear regression of genotype and GEI on environment loadings (λ_jk_), with each genotype having a distinct slope (genotypic scores, *f_ik_*) but a common intercept provided main effect of genotypes are not distinguished from GEI (Crossa, 2012). The genotypic scores measure the genotype’s sensitivity to the latent environmental factor represented by the loadings of each environment. Regardless of whether a genotype is evaluated in an environment or not, the FA model provides a predicted genetic effect for each genotype in each testing environment in the dataset.

The number of latent factors is called the order of the model and we use FAk to represent an FA model of order k. We fitted FA1 to FA4 models. The model with the minimum value of AIC was chosen as the most parsimonious model. For FAk models where *k* > 1, the matrix of loadings does not have a unique solution. Therefore, (Cullis et al., 2010) recommends rotating the estimated loadings to their principal component solution *via* singular value decomposition. We use asterisks (^*^) below to denote rotated loadings and scores.

## Assessment of overall performance and stability

We used the factor analytic selection tools proposed by (Smith and Cullis, 2018) to assess and identify the clones with high overall performance and global stability across the testing environments. If $\lambda_{1}$ represents the mean of the loadings for the first factor, then the overall performance (OP) measure for *i*th genotype is computed as


(13) $$\lambda_{1}{\tilde{f}}^{*}{}_{1i}=\frac{1}{s}\overset{s}{\underset{j=1}{\sum }}{\hat{\lambda }}^{*}{}_{1j}{\tilde{f}}^{*}{}_{1i}$$

where ${\hat{\lambda }}^{*}{}_{1j}$ is the rotated loading associated with the *j*th environment in the first latent factor, and ${\tilde{f}}^{*}{}_{1i}$ is the rotated genotypic score of the *i*th genotype in the first latent factor. The OP measure was based on the first factor loadings because they were all positive and thus represented non-crossover GE interaction (Smith and Cullis, 2018). The OP is on the same scale of measurement as the agronomic trait being analyzed.

The measure of genotype stability is usually based on the higher factors (*k* > 1) which have a mixture of both positive and negative loadings. This practice is justified by the fact that changes in genotype performance due primarily to changes in scale, which are accounted for in the first factor should be eliminated from stability analysis (Smith and Cullis, 2018). The global stability measure for each genotype was obtained as the root mean square deviation (RMSD) from the regression line associated with the first factor. The RMSD for *i*th genotype is derived as


(14) $$\sqrt{\frac{1}{s}\overset{s}{\underset{j=1}{\sum }}{\tilde{\in }}^{*2}{}_{ij}}$$

where ${\tilde{\in }}^{*2}{}_{ij}={\tilde{\beta }}_{ij}+{\hat{\lambda }}^{*}{}_{1j}{\tilde{f}}^{*}{}_{1i}$. The ${\tilde{\in }}^{*2}{}_{ij}$ denoted deviations from the first factor prediction in a plot where the x-axis was the first factor loadings and y-axis was the common effects; and ${\tilde{\beta }}_{ij}$ were the linear combination of factor loadings and genotypic scores. Like OP, RMSD is on the scale of the trait measured. The stability of the genotypes across the environments can be explored in detail by latent regression plot. In this study, we obtained the plot by regressing the predicted breeding value on the factor loading of the FA3 model.

### Clustering of target environments and locations

We used the rotated factor loadings resulting from the FA3 model for clustering and delineating the subset of environments and locations into mega-environment using the *hclust()* function in R and the Ward’s D2 linkage method. The procedure involved these steps: (i) Computing the Euclidean distance between a pair of environments from the *s* × 3 factor loadings matrix; (ii) Hierarchical clustering on the derived distance matrix using Ward’s minimum variance linkage method (ward.D2) where dissimilarities were squared before clustering; (iii) plotting and visualizing the cluster dendrogram resulting from (ii); and (iv) subjectively determining the number of clusters by imposing a threshold of minimum similarity to be in the same cluster.

To cluster locations (as opposed to environments = location-by-year combination), we computed for each factor separately, the average loadings of the environment that each location was a part of. We then used the approach above to cluster the locations.

We further used an approach proposed by Smith et al. (2021) to group the testing environments into interactive classes (iClasses), a cluster of environments where a negligible crossover GEI exist.

### Association of latent factor loadings with environmental covariables

The environmental covariables associated with GEI were identified by correlating each environmental covariable to each of the three latent factor loadings extracted from the FA3 model. Then, we fitted a partial least square regression to describe GEI in terms of differential genotypic responses to environmental covariables. The PLSR is a form of multivariate regression that maximizes covariance between *X* and *Y* data matrices in one single estimation procedure (Vargas et al., 1998). The environment covariables were in a data matrix *X* of dimension 27 × 40 (27 rows representing the testing environments and 40 columns corresponding to the environmental covariables across the developmental phases). The factor loadings were data matrix *Y* of size 27 × 3 (27 rows for testing environments and 3 columns corresponding to the latent factor loadings). Since the PLSR method is variant to the scale of measurement, the columns of *X* and *Y* data matrices were centered (zero mean) and scaled (unit variance).

The PLSR was implemented using the *plsr()* function of *pls* library (Liland et al., 2021) in R (R Core Team, 2018). The underlying multivariate PLSR in a bilinear form is described as


(15) $$X=TP^{\prime }+E$$

and


(16) $$Y=UQ^{\prime }+F$$

where *T* and *U* are, respectively, *n* × l matrix of projection of *X* (X scores) and projections of *Y* (Y scores); *P* and *Q* denote *m* × l and *p* × l orthogonal loading matrices respectively; *E* and *F* are residual matrices assumed to be independent and identically distributed random normal variables.

We recognize that in this analysis we are using the environmental loadings from FA3 model as if they were observed data as opposed to derived parameters. A better approach would have been to develop a kind of factor analytic model to work directly on the continuous environmental covariables as opposed to using environment labels as a categorical variables. We do not know of a method to do such an analysis, let alone software to fit it. We look forward to the development of such a method.

## Results

### Single-trial analysis and data quality control

Before formal statistical analysis, the distribution of observed agronomic traits of 96 clones from 48 trials tested in 28 environments revealed that the traits approximated a normal distribution across the testing environments (Supplementary Figure 2) as the mean denoted by blue data point and median represented by a line were approximately the same. The boxplots showed the heterogeneity of variation in the observed traits across the environments. The mean fresh root yield across the 48 trials varied from 0.3 t/ha (18UYT36setAKN, 18UYT36setBKN) in Kano to 83.3 t/ha (18UYT36setAOT) in Otobi with an overall mean of 27.6 t/ha (Supplementary Table 1). The broad-sense heritability on an entry-mean basis (H^2^) ranged from 0.06 (19UYT36setAMK) to 0.85 (18UYT36setBIK) across trials. We observed experimental accuracy (Ac) values varying from 0.24 (19UYT36SETAMK) to 0.91 (18UYT36setBIK). The coefficients of variation (CV%) ranged from 14% (17UYT36setAIK) to 42% (18UYT36setAKN). The four trials (17C1UYT34UM, 18UYT36setAKN, 19UYT36setAZA, and 19UYT36setAMK) displayed in red (Supplementary Figures 3a,b) were filtered out from the combined analysis based on threshold defined in the Methods because their error variances were in the range of 17 to 30 fold higher than the genetic variances, which was very unusual in our breeding program. Therefore, subsequent analysis was based on 44 trials across 27 environments.

### Variance–covariance structure model

The pair of environments with the least connectivity had five clones in common while Ikenne18 and Mokwa18 had 96 in common (Supplementary Table 2). The low or poor connectivity between some pairs of environments may impact the reliability of estimation of between environment genetic covariances (Smith et al., 2015). Note, however, that because we used an HRM between clones, relationship among clones in a pair of environments helps increase the accuracy of covariance estimation between the pair.

The diagonal variance model revealed that genetic variance within environments ranged from 2.2 (Zaria20) to 82.3 (Ikenne20) under the assumption that genetic correlation between pairs of environments was zero (Supplementary Table 3). The compound symmetry model showed a uniform genetic correlation of 0.42 corresponding to the uniform genetic variance of 22.3 within environments (Supplementary Table 4). The compound symmetry heterogeneous model estimated a uniform genetic correlation of 0.53 but unique genetic variance within environments resulting in different genetic covariances between pair of environments (Supplementary Table 5).

We reported the total number of parameters, the model log-likelihood (Loglik), Akaike information criterion (AIC), Bayesian information criterion (BIC) and percentage of genetic variance captured by factor analytic models (Table 2). The first two ranking models were FA3 and FA4 models having AIC values of 20338.3 and 20339.8, respectively, (Table 2). The FA3 model was chosen as the optimal model because it had the lowest AIC. It required 152 parameters to capture 79.0% of genotypic effect within environments (Table 2).

**Table 2:** Summary of the models fitted to the combined MET data set.

Model	Parameter	LogLik	AIC	BIC	Var (%)
DIAG	89	−10255.7	20689.4	21250.7	
CS	55	−10188.2	20486.3	20833.2	
CSH	79	−10109.0	20377.1	20875.3	
FA1	107	−10078.3	20370.7	21045.5	57.2
FA2	128	−10043.6	20343.2	21150.4	70.8
FA3	152	−10017.3	20338.3	21296.8	79.0
FA4	170	−9999.9	20339.8	21411.9	83.3

Presented is the number of variance–covariance parameters, residual log-likelihood (LogLik), AIC, Akaike information criterion and BIC, Bayesian information criterion, and the mean percentage of variance accounted for. DIAG Diagonal variance model; CS, Compound symmetry model; CSH, Compound symmetry heterogeneous model; and FAk: Factor analytic model of order *k*.

**Figure 2:**
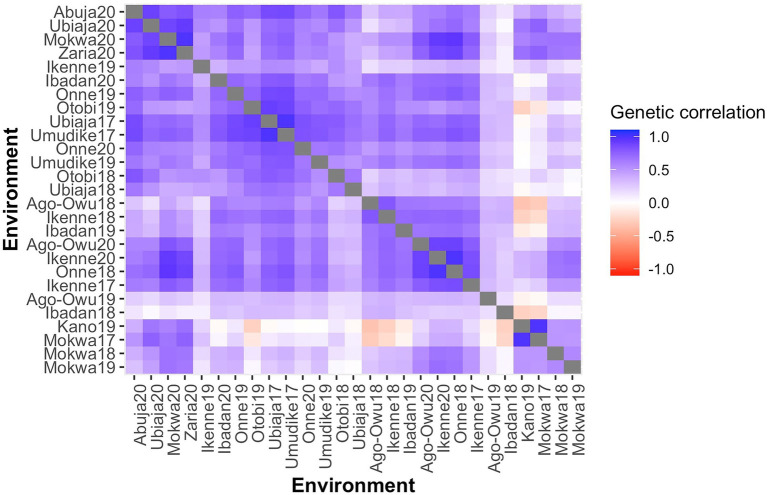
A Heatmap of pairwise genetic correlations of fresh root yield estimated the from FA3 model for 27 environments, ordered based on the dendrogram of Ward’s D2 linkage method. The color of the square is related to the magnitude of the genetic correlation between environments.

Pairwise genetic correlations among environments, as estimated by the FA3 model, were predominantly positive (Figure 2), varying from −0.34 (Ago-owu18 vs. Kano19) to 1.00 (Umudike17 vs. Ubiaja17). We report the estimated genetic correlations, variances, and covariances among the environments (Supplementary Table 6). Genetic correlations estimated above 0.70 between any pair of environments were considered high and equivalent to low GEI: the genotypes exhibited similar fresh root yield performance between such environments. In contrast, pairs of environments showing correlations below 0.40, indicated high GEI: the genotypes ranked differently across these pairs of environments.

### Rotated factor loadings

The first factor loadings after rotation to the principal component solution were all positive, indicating non-crossover GEI, varying from 0.3 to 8.6 with a median of 3.2 and a mean of 3.6 (Table 3). The remaining two factors had ranges extending into negative values indicating crossover GEI. The first-three factors jointly explained 79.0% of the environments’ total genetic variability such that the first, second and third factors accounted for 53.5, 14.0, and 11.5% of total genetic variability, respectively, (Table 3). The heritability resulting from genetic and error variances of FA3 model ranged from 0.09 (Kano19) to 0.59 (Ikenne17 and Ikenne18) with an average value of 0.39 across the environments (Table 3).

**Table 3:** Summary of the FA3 model in terms of factor loadings, specific variance (Ψ) and genetic variances$\;\left(\sigma_{g}^{2}\right)$, error variances ($\sigma_{e}^{2}$), heritability (H^2^), and interactive classes (iClasses) for environment.

Environment	Factor loadings			Variances			H^2^	iClasses
	Factor 1	Factor 2	Factor 3	Ψ	$\sigma_{g}^{2}$	$\sigma_{e}^{2}$		
Abuja20	3.5	0.3	−2.8	1.3	21.1	35.1	0.37	ppn
Ago-Owu18	3.4	1.3	2.1	5.4	22.0	47.5	0.32	ppp
Ago-Owu19	1.8	0.4	0.5	2.6	20.5	45.6	0.31	ppp
Ago-Owu20	6.2	−0.9	1.0	8.7	47.5	53.6	0.47	pnp
Ibadan18	2.2	1.8	1.4	0.0	48.4	67.6	0.42	ppp
Ibadan19	5.6	0.9	2.0	11.1	57.7	60.8	0.49	ppp
Ibadan20	3.2	0.7	0.2	4.7	14.7	43.4	0.25	ppp
Ikenne17	7.8	−0.6	0.3	0.0	80.3	54.7	0.59	pnp
Ikenne18	5.5	1.7	2.4	5.6	44.2	30.7	0.59	ppp
Ikenne19	1.7	0.4	−1.7	8.7	14.0	21.4	0.40	ppn
Ikenne20	8.6	−2.9	1.2	0.0	81.8	58.2	0.58	pnp
Kano19	0.3	−1.5	−0.9	0.0	2.8	28.5	0.09	pnn
Mokwa17	1.1	−3.5	−2.7	0.0	19.8	23.7	0.45	pnn
Mokwa18	3.8	−3.4	0.1	1.2	48.4	51.3	0.49	pnp
Mokwa19	2.9	−3.1	0.7	10.8	28.0	33.0	0.46	pnp
Mokwa20	2.9	−1.3	−0.7	0.0	10.0	10.1	0.50	pnn
Onne18	4.7	−1.3	0.3	0.0	23.1	81.4	0.22	pnp
Onne19	3.1	0.5	−0.6	0.0	12.2	15.6	0.44	ppn
Onne20	1.7	0.7	−0.6	1.6	5.0	14.1	0.26	ppn
Otobi18	2.4	1.5	−2.6	0.0	18.5	52.8	0.26	ppn
Otobi19	5.2	4.0	−1.5	5.4	48.7	122.7	0.28	ppn
Ubiaja17	5.0	2.0	−1.4	0.0	29.4	34.5	0.46	ppn
Ubiaja18	2.2	1.6	−1.7	0.0	17.7	23.0	0.43	ppn
Ubiaja20	2.8	−1.1	−2.4	0.6	14.9	15.0	0.50	pnn
Umudike17	3.5	1.1	−0.9	0.0	13.6	36.2	0.27	ppn
Umudike19	4.5	1.0	−0.4	11.2	31.3	61.5	0.34	ppn
Zaria20	1.5	−0.8	−0.6	0.0	3.0	12.9	0.19	pnn
Min	0.3	−3.5	−2.8	0.0	2.8	10.1	0.09	
Max	8.6	4.0	2.4	11.2	81.8	122.7	0.59	
Median	3.2	0.4	−0.6	0.6	21.0	36.2	0.42	
Mean	3.6	0.0	−0.3	2.9	28.8	42.0	0.39	

### Assessment of overall performance and stability

**Figure 3:**
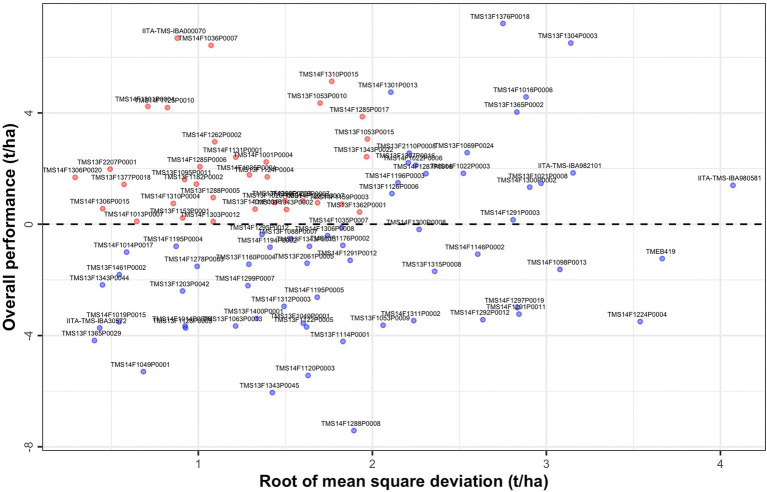
Overall performance (OP) vs. stability (Root of mean square deviation, RMSD) for fresh root yield showing all 96 clones evaluated across the environments.

The characteristics of first and higher factor loadings can be used to determine the overall performance (OP) and stability of the genotypes. A scatter plot of the OP against the root mean square deviation (RMSD) visualizes genotype performance and its stability (Figure 3). Genotypes in the top left-hand side of the plot had high performance and stability while those in the bottom right-hand side had low performance and stability (see also Supplementary Table 7).

Genotype stability may be best viewed using latent regression plots which revealed genotypic responses to each factor loading (Smith et al., 2015). We considered latent regression plots for six clones which included the top two overall performance clones (TMS13F1376P0018 and IITA-TMS-IBA000070), top two stability clones (TMS14F1306P0020 and TMS13F1365P0029), and two clones known for possessing high industrial starch content (TMEB419 and TMS14F1036P0007; Supplementary Figures 4–6). Regression lines have slopes given by the estimated genotype scores for the individual and factor concerned. The regression on the first factor has a maximum impact on the predicted breeding values for explaining the largest percentage (53.5%) of total genetic variation. Since the estimated loadings for this factor are non-negative, large positive regression coefficients for this factor indicate high fresh root yield.

### Clustering of target environments and locations

**Figure 4:**
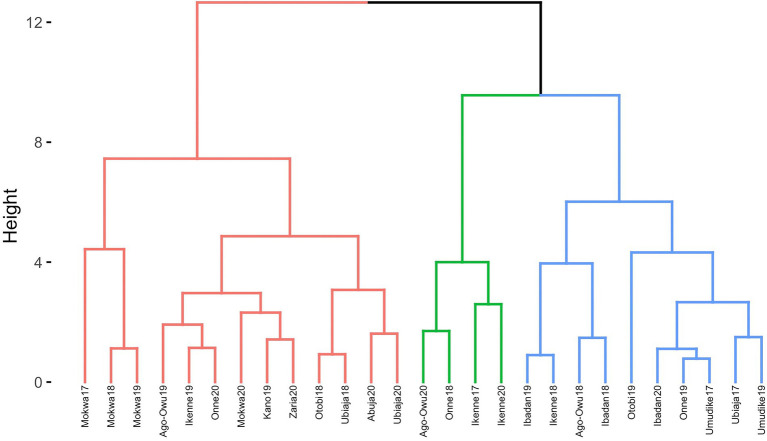
Dendrogram of 27 environments based on cassava fresh root yield using rotated factor loadings from FA3 model and Ward’s D2 linkage method.

**Figure 5:**
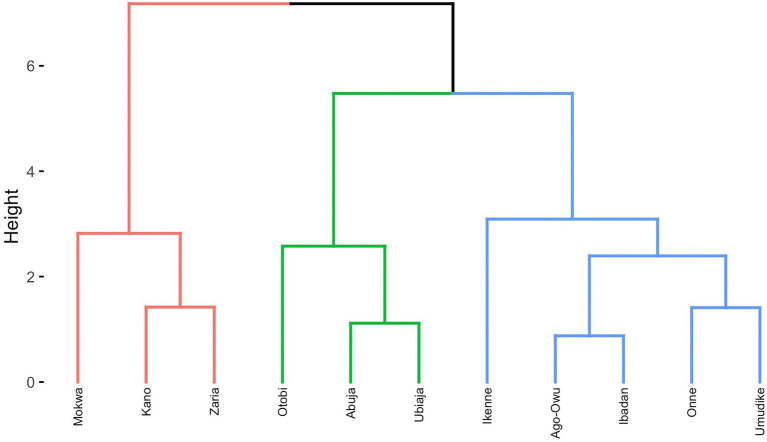
Dendrogram of 11 locations based on cassava fresh root yield using average rotated factor loadings from FA3 model and Ward’s D2 linkage method.

Dendrogram clusters of 27 environments (Figure 4) and 11 locations (Figure 5) using the loadings from the FA3 model reflected how the environments and locations were related. The environments were clustered at a distance of approximately eight while the locations were grouped at distance height of approximately three. This is an indication that, averaged over years, locations are less differentiated than environments. There was a consistent pattern of Mokwa belonging to the same cluster with environments Kano and Zaria over years (Figures 4, 5). Likewise, the environments associated with Ikenne are in the same cluster except for Ikenne19 which belonged to another cluster. We observed consistent similarity in the environments of Ago-Owu, Onne, Ikenne, and Ibadan, so that these locations were also clustered (Figures 4, 5). The environments Umudike17, Umudike19 shared common characteristics with one out of the three environments in Onne (Figure 4) leading those two locations to be clustered (Figure 5). We identified four interactive classes (pnn, pnp, ppn, and ppp) of the possible 2^3^ = 8 iClasses with 5, 6, 10, and 6 environments each (Table 4). Each of these clusters of environments had a minimal crossover genotype-by-environment interaction and the contrasts between the environments within the same cluster group were eliminated (Smith et al., 2021).

**Table 4:** Mean factor loadings, number and name of environments within each of four interactive classes (iClasses).

iClass	Factor 1	Factor 2	Factor 3	Number of environment	Environment
pnn	1.7	−1.6	−1.5	5	Kano19, Mokwa17, Mokwa20, Ubiaja20, Zaria20
pnp	5.7	−2.0	0.6	6	Ago-Owu20, Ikenne17, Ikenne20, Mokwa18, Mokwa19, Onne18
ppn	3.3	1.3	−1.4	10	Abuja20, Ikenne19, Onne19, Onne20, Otobi18, Otobi19, Ubiaja17, Ubiaja18, Umudike17, Umudike19
ppp	3.6	1.1	1.4	6	Ago-Owu18, Ago-Owu19, Ibadan18, Ibadan19, Ibadan20, Ikenne18

pnn, positive negative negative; pnp, positive negative positive; ppn, positive positive negative; and ppp, positive positive positive.

### Association of factor loadings with environmental covariables

The first PLSR component had relatively high positive X-loadings for environmental covariables TRAN1, TRAN2, TRAN3, TRAN4, TMAX1, TMAX2, TMAX3, TMAX4, SRAD1, SRAD2, SRAD3, and SRAD4 (Table 5) and showed high negative Pearson’s correlation coefficients (*r*) with the first factor loading of the FA3 model. However, these environmental covariables were in contrast to RH1, RH2, RH3, RH4, RZSW1, RZSW2, RZSW3, RZSW4, SM1, SM2, SM3, SM4, SSW1, SSW2, SSW3, SSW4, and TMIN2 showing high negative X-loadings in the first PLSR component and positively correlated to first factor loading. Conversely, the second PLSR component identified WS1, WS2, WS3, and WS4 as environmental covariables that had moderately high negative X-loadings.

**Table 5:** X-loadings of the first and second PLSR components of environmental covariables and their Pearson’s correlation coefficients sorted in descending order of the first latent factor loadings extracted from the FA3 model.

Environmental covariables	Partial least squares		Factor analytic model		
	Component 1	Component 2	Factor 1	Factor 2	Factor 3
RH3	−0.19	0.07	0.45	0.48	0.45
SM3	−0.19	−0.04	0.42	0.43	0.25
RH2	−0.19	0.03	0.41	0.28	0.35
SM2	−0.20	−0.09	0.40	0.43	0.19
SM1	−0.20	−0.04	0.40	0.49	0.31
TMIN2	−0.16	0.05	0.39	0.10	0.25
RH4	−0.18	−0.17	0.37	0.40	0.17
RH1	−0.18	−0.19	0.36	0.50	0.08
SSW3	−0.20	0.05	0.36	0.41	0.36
RZSW1	−0.20	−0.06	0.36	0.47	0.29
SM4	−0.20	−0.10	0.36	0.48	0.26
RZSW3	−0.19	0.02	0.35	0.42	0.32
SSW1	−0.20	−0.09	0.35	0.51	0.24
SSW2	−0.19	−0.03	0.35	0.37	0.24
RZSW2	−0.19	−0.02	0.33	0.33	0.23
SSW4	−0.19	−0.12	0.28	0.44	0.23
RZSW4	−0.19	−0.12	0.27	0.46	0.23
TMIN3	−0.11	−0.06	0.25	0.34	0.10
PRECIP3	−0.07	0.31	0.14	0.21	0.53
WS1	0.01	−0.43	0.10	0.09	−0.40
TMIN1	−0.08	0.05	0.08	−0.05	0.05
PRECIP4	−0.07	0.07	0.06	0.25	0.30
PRECIP1	−0.11	0.08	0.02	0.20	0.08
TMIN4	0.00	0.04	−0.05	−0.21	−0.18
PRECIP2	−0.10	0.13	−0.05	0.04	0.11
WS4	0.09	−0.41	−0.16	0.03	−0.45
SRAD2	0.16	−0.04	−0.21	−0.11	−0.32
WS3	0.12	−0.41	−0.23	−0.03	−0.48
WS2	0.10	−0.39	−0.24	−0.12	−0.41
TMAX2	0.17	−0.03	−0.24	−0.34	−0.29
SRAD3	0.18	−0.13	−0.27	−0.38	−0.40
TRAN4	0.18	0.14	−0.31	−0.35	−0.13
TMAX4	0.17	0.15	−0.31	−0.42	−0.20
TRAN1	0.18	0.22	−0.31	−0.36	−0.01
SRAD4	0.16	0.06	−0.32	−0.52	−0.25
TMAX1	0.17	0.28	−0.32	−0.44	0.01
TMAX3	0.18	−0.15	−0.32	−0.43	−0.51
SRAD1	0.17	0.07	−0.33	−0.24	−0.08
TRAN2	0.18	−0.05	−0.36	−0.23	−0.30
TRAN3	0.18	−0.07	−0.37	−0.49	−0.40

TMAX, mean maximum temperature; TMIN, mean minimum temperature; TRAN, mean temperature range; PRECIP, total precipitation; RH, mean relative humidity; WS, mean wind speed; SRAD, mean solar radiation; SSW, mean surface soil wetness; RZSW, mean root zone soil wetness; SM, mean soil moisture. The suffixes 1, 2, 3, and 4 denote the covariables measured at first, second, third, and fourth developmental phases of cassava crop, respectively.

Based on Pearson’s correlation coefficients, we observed that RH2, RH3, SM1, SM2, and SM3 were weather conditions that had high positive association to factor 1 but in contrast to TRAN1, TRAN2, TRAN3, TRAN4, TMAX1, TMAX3, TMAX4, SRAD1, and SRAD4 which revealed high negative correlation (Table 5); SSW1, RH1, RH3, SM1, SM4, RZSW1, RZSW4 were positive highly correlated but in contrast to SRAD4, TRAN3, TMAX1, TMAX3, TMAX4 which showed high negative correlation to factor 2; and PRECIP3, PRECIP4, RH2, RH3, RZSW3, SM1, and SSW3 had positive association but contrary to WS1, WS2, WS3, WS4, SRAD3, TRAN3, and TMAX3 (in factor 3) affected genotypic responses within environments clustered by these three factors. This analysis provides useful information to understand the environmental covariates’ influence on the clonal performance across the environments. It further allows the identification of the most likely environmental conditions affecting GEI in the testing environments.

**Figure 6:**
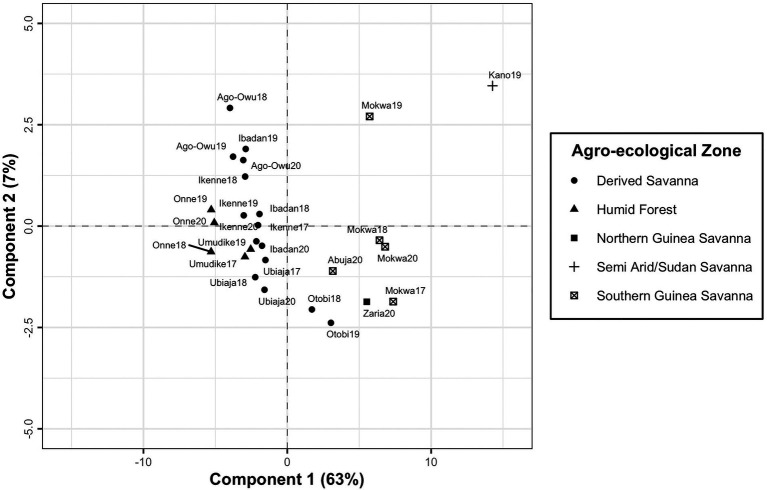
A plot of first and second components of X-scores revealing the grouping of the testing environments based on latent factor loadings from FA3 model and environmental covariables.

PLSR was used to maximize covariance between the factor loadings and the environmental covariables at four developmental phases of cassava root crop. It identified the most significant environmental conditions influencing differential genotypic yield response in the testing environments. The first latent component resulting from fitting PLSR model explained 63% of variance in factor loadings. The addition of second component resulted in capturing 70% of total variation, and after the third component which accounted for 78% of variation, no significant improvement in the variance explained in the factor loadings. It was revealed that the first component separated the environments into two clustered groups and conversely its second component did not have a clear interpretation (Figure 6). The environment Kano19 was identified as a leverage point well separated from other environments (Figure 6).

**Figure 7:**
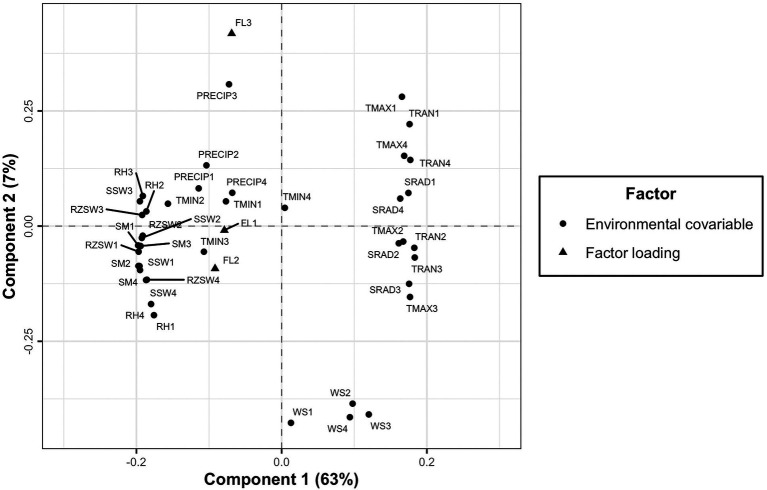
A plot of X and Y loadings revealing the association of factor loadings resulting from FA3 model to environmental covariables across four developmental phases of cassava.

However, a PLSR biplot of *X* and *Y* loadings revealed the association between environmental covariables at different developmental phases and factor loadings. The second component separated the third factor loading (FL3) from the remaining two factor loadings (FL1 and FL2; Figure 7). The environmental covariables found close to each other or in the close vicinity of factor loadings were positively correlated to each other and those situated in the opposite side are negatively correlated (Figure 7).

## Discussion

The IITA cassava breeding program continually evaluates many clones in several target locations over years aiming to identify clones with high yield productivity and stability and to assess adaptability across a wide range of diverse environmental conditions. This evaluation necessitates the establishment of multi-environment trials annually to determine the clones’ yield performance across various agro-ecological zones in Nigeria. The release of new cultivars arises when the clones possess specific characteristics that prove their desirable performance for a given geographical region, emphasizing the importance of MET in plant breeding programs (Oliveira et al., 2020).

Studying the patterns of MET data for decision making cannot be adequately investigated using conventional statistical methods due to some limitations as pointed out by Bakare et al. (2022). Therefore, factor analytic structures fitted in the linear mixed model framework as used in this study are flexible and robust for modeling complex genetic variance structure and more parsimonious for MET analyses than unstructured models (Smith et al., 2001a). Linear mixed models show great flexibility in handling unbalanced data that occur in METs due to unforeseen circumstances. The analyses of MET data have been broadly implemented using FA structures to understand the stability and adaptability of genotypes across testing environments (Li et al., 2017; Dias et al., 2018), and also to delineate mega-environments in plant breeding (Smith et al., 2015; Monteverde et al., 2018; Smith and Cullis, 2018).

Our study is the first to implement the FA model and to identify the factors influencing GEI in cassava. Furthermore, to our awareness, this is the first study that explored the extent of association between environmental covariables and factor loadings to examine the potential factors influencing GEI for fresh root yield in cassava. In this study, the Pearson’s correlation between the environmental covariables and the factor loadings was used to describe the likely factors affecting GEI, as proposed by (Sae-Lim et al., 2014). This information is helpful to ascertain the effect each covariable has on genotypic performance across environments, toward identifying the most likely covariables affecting GE in a given set of environments. In general, relative humidity and temperature were the environmental covariates that explained the most genetic variability of fresh root yield across the environments. This information can support the breeders in recommending cassava clones for particular environments based on environmental covariables observed there historically. This ability will also facilitate the optimization of the number of testing environments for late stages of the breeding program, prioritizing environments with diverse environmental conditions.

The PLSR approach was found to be effective in clustering the testing environments from the southern region separately from that of the northern region of Nigeria based on factor loadings and environmental covariables incorporated into the model. The *X* and *Y* loadings biplot (Figure 7) showed that the GEI in the southern part of Nigeria was driven mostly by weather conditions such as minimum temperature, relative humidity, precipitation, surface soil wetness, root zone soil wetness, and soil moisture across the developmental phases of cassava. However, differential genotypic sensitivity across the environments in the north of Nigeria was mostly determined by wind speed, maximum temperature, temperature range and soil radiation. This study was limited to the environments where cassava breeders operate in Nigeria. The findings from PLSR could be used to restructure Nigerian breeding programs and adjust evaluation locations accordingly. However, future studies should explore how the environmental covariates could be used to forecast the performance of cassava clones in locations that were not within those evaluated in the previous MET.

The iClasses and ward.D2 hierarchical cluster were two approaches used to group the environments using the factor loadings. The former identified 4 clusters of environments based on the positive or negative signs of the loadings. Meanwhile the latter classified the environments into 3 cluster groups in terms of minimizing the change in variance. The two approaches showed a degree of similarity in terms of clustering environments from the same geographical regions together.

The use of latent regression plots to study yield stability and adaptability of genotypes across testing environments was recommended (Smith et al., 2015). In their approach, the predicted breeding values of genotypes are regressed on the factor loadings of the FA model. This study used FA structures and latent regression plots to identify cassava clones with high overall performance (TMS13F1376P0018 and IITA-TMS-IBA000070) and stability (TMS14F1306P0020 and TMS13F1365P0029) with their respective predicted genotypic scores for the first three factors (Supplementary Table 7).

## Conclusion

This study demonstrated that the factor analytic model was the most parsimonious variance model to dissect and account for complex patterns of GEI by separating genetic effects into common and specific variance components. The delineation of testing environments or locations into clusters through a factor analytic model was an efficient way to optimize the resources by using one location per cluster group. The use of partial least squares regression proved to be an effective tool for identifying relevant environmental covariables affecting differential genotypic sensitivity in the context of multi-environment trials where a number of external environmental covariables are incorporated in modeling. Among the environmental covariables explored in this study, minimum temperature, precipitation, relative humidity, surface soil wetness, root zone soil wetness, and soil moisture were identified as the strongest influence on genotypic responses across the testing environments in the southern region of Nigeria. This was in contrast to maximum temperature, wind speed, and temperature range (difference between maximum and minimum temperature), and solar radiation affecting GEI in the northern region of Nigeria.

## Data availability statement

The datasets presented in this study can be found in online repositories. The names of the repository/repositories and accession number(s) can be found at: https://github.com/mab658/variance_covariance_model_GxE.

## Author contributions

MB, IR, J-LJ, CE, and PK: design and study conceptualization. IR, MB, SK, CA, and EP: study methodology and implementation. MB, SK, MW, J-LJ, and IR: formal data curation, analysis, and manuscript drafting. MB, SK, CA, MW, J-LJ, PK, and IR: manuscript reviewing and editing. IR, J-LJ, and PK: supervision, coordination and funding acquisition. All authors contributed to the article and approved the submitted version.

## Funding

The research reported in this manuscript was generously funded by the Bill & Melinda Gates Foundation (https://www.gatesfoundation.org) and the United Kingdom’s Foreign, Commonwealth & Development Office (FCDO https://www.gov.uk/government/organisations/foreign-commonwealth-development-office) through the “Next Generation Cassava Breeding project” (https://www.nextgencassava.org; grant no. INV-007637) managed by Cornell University.

## Conflict of interest

The authors declare that the research was conducted in the absence of any commercial or financial relationships that could be construed as a potential conflict of interest.

## Publisher’s note

All claims expressed in this article are solely those of the authors and do not necessarily represent those of their affiliated organizations, or those of the publisher, the editors and the reviewers. Any product that may be evaluated in this article, or claim that may be made by its manufacturer, is not guaranteed or endorsed by the publisher.

## Footnotes

^1^ https://power.larc.nasa.gov/data-access-viewer/
