# Supplementary material for: Parsimonious genotype by environment interaction covariance models for cassava (Manihot esculenta)
Source: Front Plant Sci. 2022 Sep 21;13:978248. doi: 10.3389/fpls.2022.978248 (PMC9532941; doi:10.3389/fpls.2022.978248)
Supplement: Supplementary file 1 [file Data_Sheet_1.PDF]

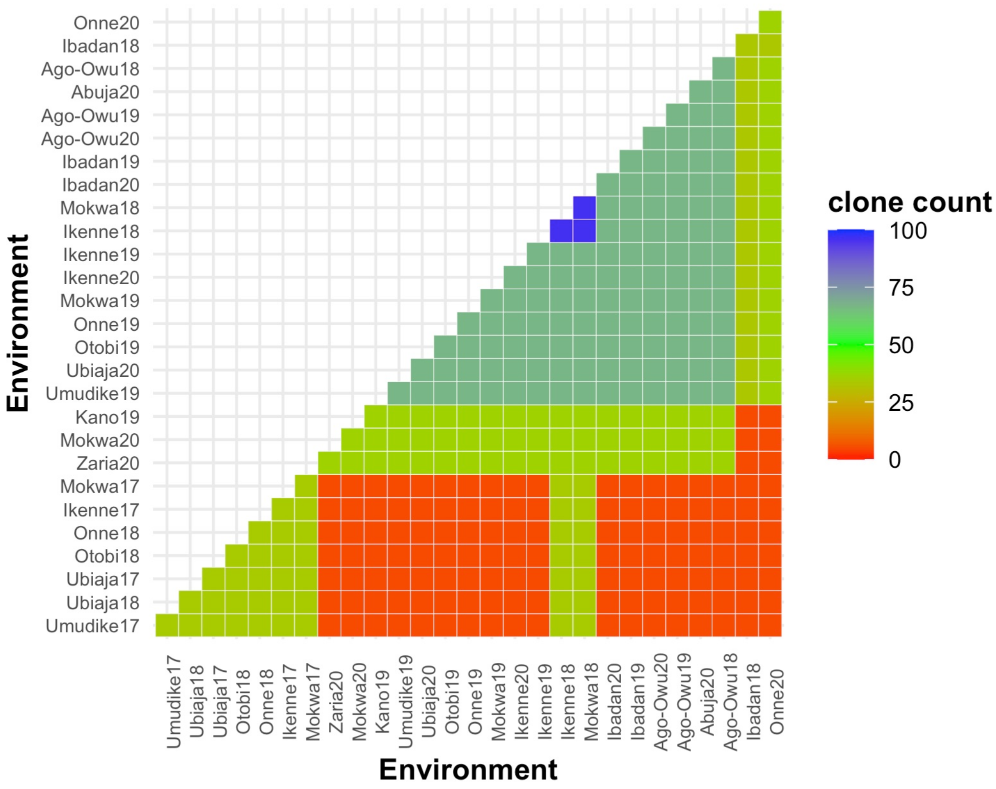

Supplementary Figure 1. A heatmap showing the clone connectivity across environments. Boxes along the diagonal give the number of clones evaluated within an environment. Off-diagonal boxes denote the number of clones in common between pairs of environments.

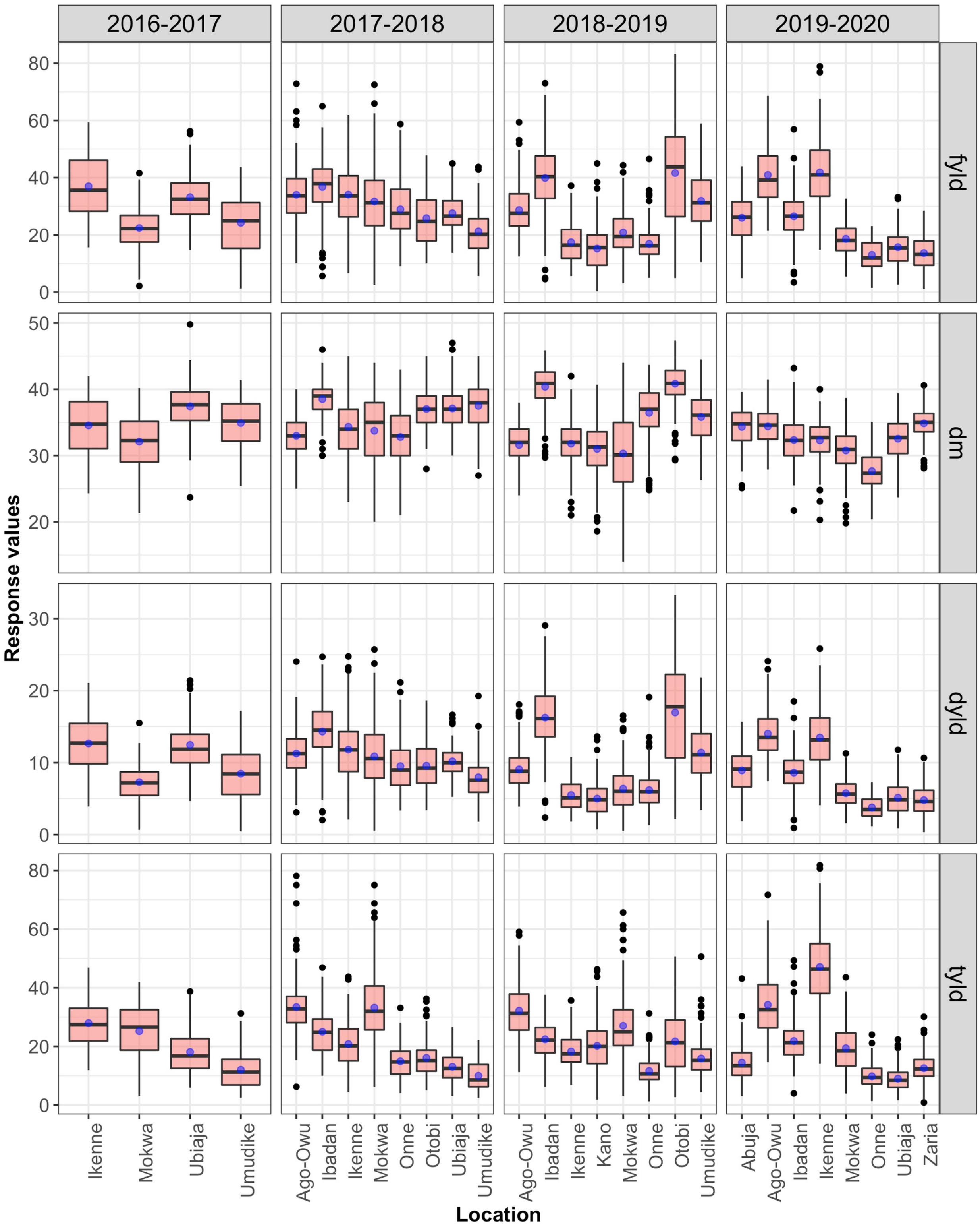

Supplementary Figure 2. Box plot displaying the distribution of fresh root yield (fyld t/ha), dry matter content (dm %), dry yield (dyld t/ha), and top yield (tyld t/ha) of 96 clones tested in 48 trials over 28 environments (location x year combination) in terms of total range, interquartile range (box), median (line), and mean (blue data point).

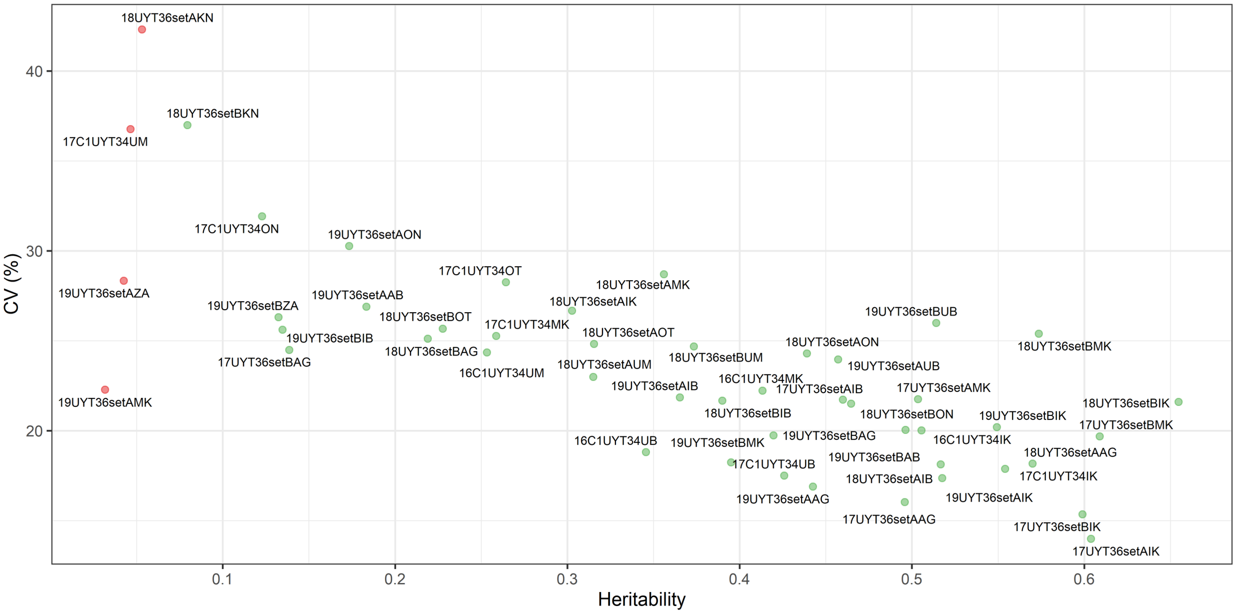

Supplementary Figure 3a. Scatter plot of cofficient of correlation (CV %) versus heritability assessing data quality of individual trials for fresh root yield (t/ha). Trials whose CV above 40.5 or heritability below 0.05 displayed in a red data point were removed from combined analysis.

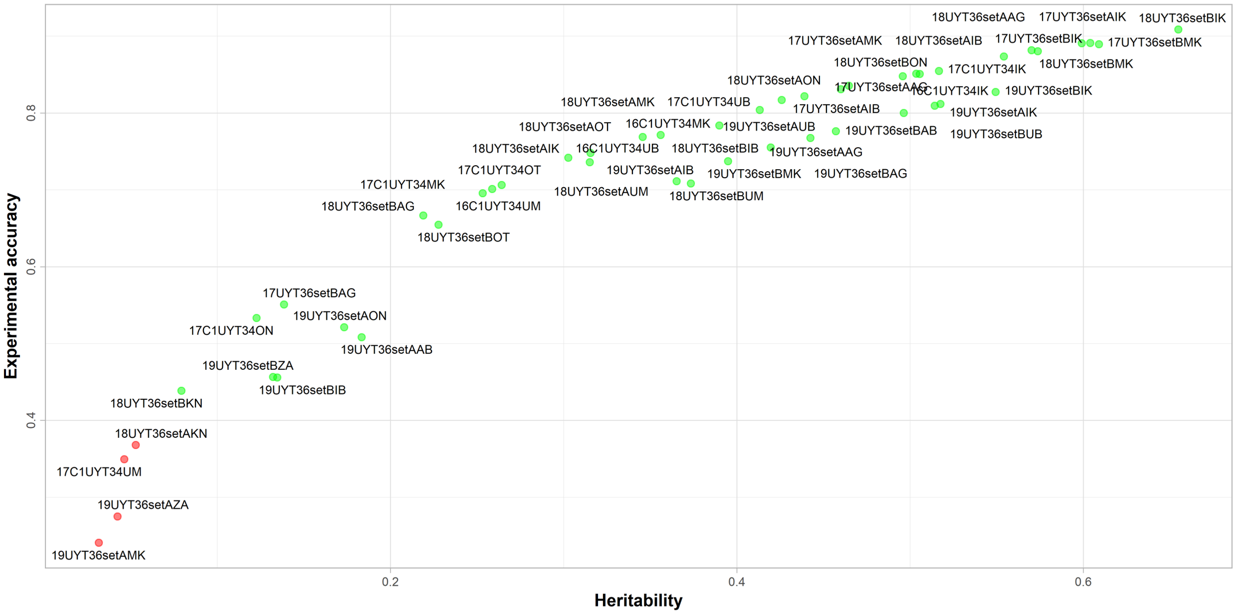

Supplementary Figure 3b. Scatter plot of experimental accuracy versus heritability assessing data quality of individual trials for fresh root yield (t/ha). Trials whose Accuracy below 0.40 or heritability below 0.05 displayed in a red data point were removed from combined analysis.

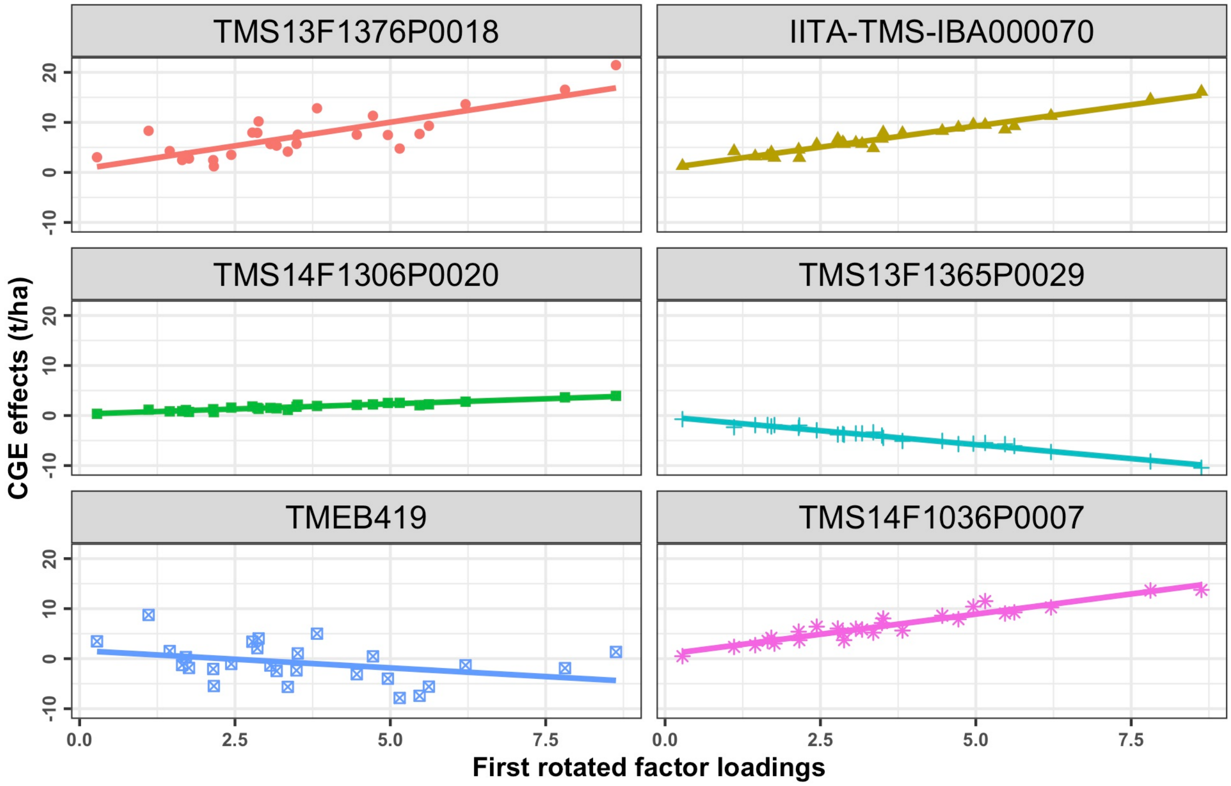

Supplementary Figure 4. Latent genetic regression plot for the first rotated factor for six genotypes, including the top 2 overall performances, the top 2 stable genotypes, and the two genotypes TMEB419 and TME14F1036P0007 known for industrial starch content

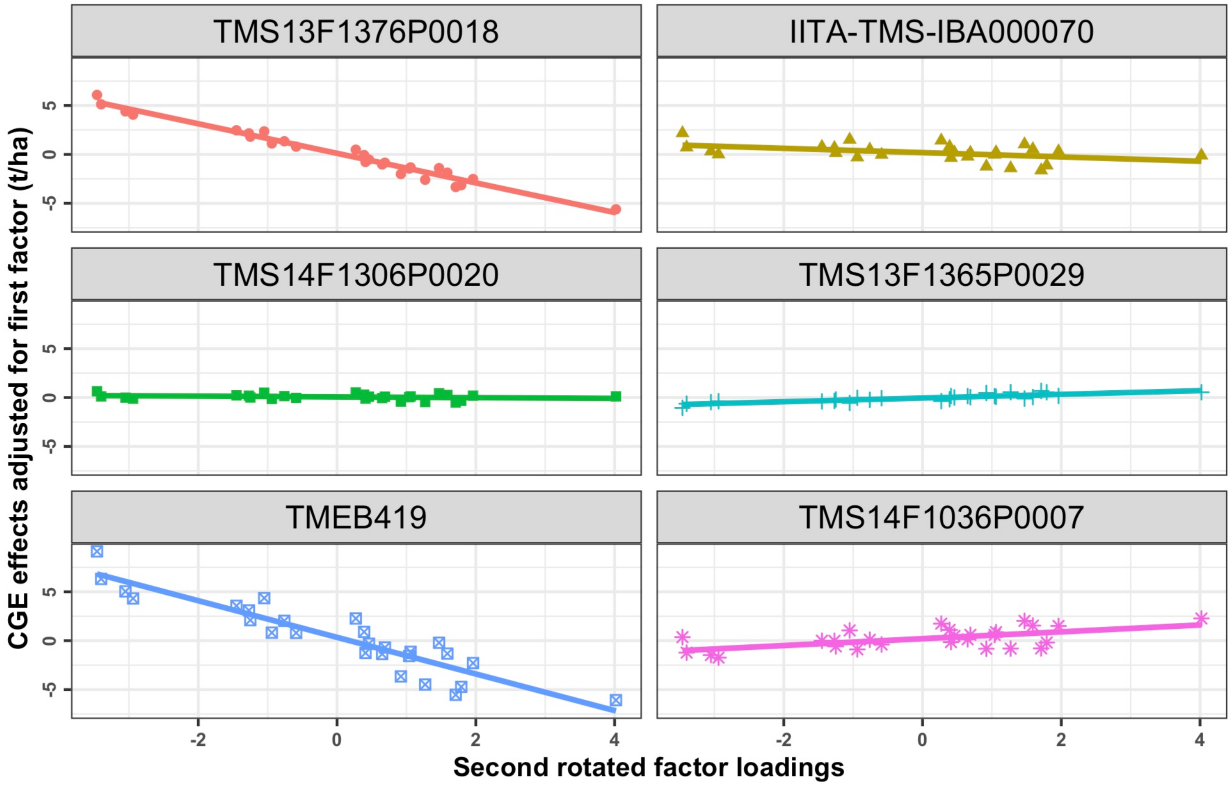

Supplementary Figure 5. Latent genetic regression plot for the second rotated factor for six genotypes which includes top 2 overall performances, top 2 stable, and the two genotypes TMEB419 and TME14F1036P0007 known for industrial starch content.

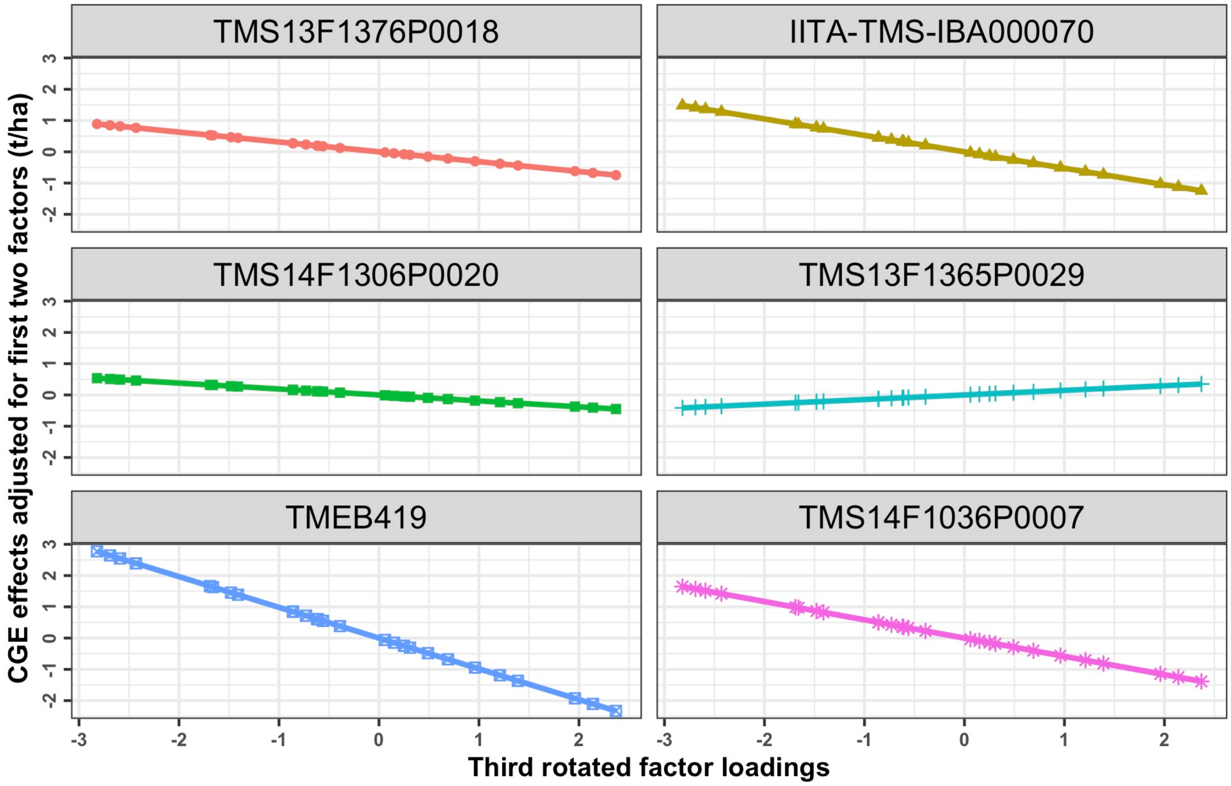

Supplementary Figure 6. Latent genetic regression plot for the third rotated factor for six genotypes, including the top 2 overall performances, the top 2 stable, and the two genotypes TMEB419 and TME14F1036P0007 known for industrial starch content.
